# A Cell‐free DNA Barcode‐Enabled Single‐Molecule Test for Noninvasive Prenatal Diagnosis of Monogenic Disorders: Application to β‐Thalassemia

**DOI:** 10.1002/advs.201802332

**Published:** 2019-04-01

**Authors:** Xingkun Yang, Qinghua Zhou, Wanjun Zhou, Mei Zhong, Xiaoling Guo, Xiaofeng Wang, Xin Fan, Shanhuo Yan, Liyan Li, Yunli Lai, Yongli Wang, Jin Huang, Yuhua Ye, Huaping Zeng, Jun Chuan, Yuanping Du, Chouxian Ma, Peining Li, Zhuo Song, Xiangmin Xu

**Affiliations:** ^1^ Department of Medical Genetics School of Basic Medical Sciences Southern Medical University Guangzhou Guangdong 510515 China; ^2^ Guangdong Technology and Engineering Research Center for Molecular Diagnostics of Human Genetic Diseases Guangzhou Guangdong 510515 China; ^3^ Affiliated Foshan Maternity & Child Healthcare Hospital Southern Medical University Foshan Guangdong 528000 China; ^4^ Guangdong Key Laboratory of Biological Chip Guangzhou Guangdong 510515 China; ^5^ The Center for Precision Medicine of First Affiliated Hospital Biomedical Translational Research Institute School of Pharmacy Jinan University Guangzhou Guangdong 510632 China; ^6^ Hunan Research Center for Big Data Application in Genomics Genetalks Inc. Changsha Hunan 410152 China; ^7^ Nanfang Hospital Southern Medical University Guangzhou Guangdong 510515 China; ^8^ Guangxi Zhuang Autonomous Region Women and Children Care Hospital Nanning Guangxi 530000 China; ^9^ Qinzhou Maternity & Child Healthcare Hospital Qinzhou Guangxi 535000 China; ^10^ Department of Genetics Yale University New Haven CT 06520 USA

**Keywords:** β‐thalassemia, cell‐free DNA barcode‐enabled single‐molecule test (cfBEST), molecule counting system, monogenic disorders, noninvasive prenatal diagnosis (NIPD)

## Abstract

Noninvasive prenatal testing of common aneuploidies has become routine over the past decade, but testing of monogenic disorders remains a challenge in clinical implementation. Most recent studies have inherent limitations, such as complicated procedures, a lack of versatility, and the need for prior knowledge of parental genotypes or haplotypes. To overcome these limitations, a robust and versatile next‐generation sequencing‐based cell‐free DNA (cfDNA) allelic molecule counting system termed cfDNA barcode‐enabled single‐molecule test (cfBEST) is developed for the noninvasive prenatal diagnosis (NIPD) of monogenic disorders. The accuracy of cfBEST is found to be comparable to that of droplet digital polymerase chain reaction (ddPCR) in detecting low‐abundance mutations in cfDNA. The analytical validity of cfBEST is evidenced by a β‐thalassemia assay, in which a blind validation study of 143 at‐risk pregnancies reveals a sensitivity of 99.19% and a specificity of 99.92% on allele detection. Because the validated cfBEST method can be used to detect maternal‐fetal genotype combinations in cfDNA precisely and quantitatively, it holds the potential for the NIPD of human monogenic disorders.

## 1 Introduction

Aneuploidies and disease‐causing mutations are detected both invasively and noninvasively in current prenatal routine tests. The inherent shortcomings of invasive procedures, which include added anxiety to pregnant women and an increased risk of miscarriage or injury to fetuses, prompted the advent and ongoing development of noninvasive tests. The discovery of cell‐free fetal DNA (cffDNA) in maternal circulation ushered in a new era of noninvasive prenatal testing (NIPT).^1^ The presence of cffDNA in maternal blood provides an accessible noninvasive biomarker originating from fetuses; the next‐generation sequencing (NGS) made it possible to pinpoint the relative dosage of each chromosome precisely for screening chromosome aneuploidies.^2^, ^3^, ^4^ The right technology being applied to the right targets led to the worldwide success of NIPT on aneuploidies, which was rapidly incorporated into routine clinical practice.

For monogenic disorders, different noninvasive prenatal diagnosis (NIPD) strategies have been presented. Because cffDNA exists in the background of maternal cfDNA, earlier studies, such as the diagnosis of autosomal dominant disorders,^5^ paternally inherited genetic disorders like rhesus D,^6^ and the exclusion of compound heterozygotes of autosomal recessive disorders like β‐thalassemia,^7^, ^8^ focused on genotyping paternally originated alleles. These pioneering studies provided proof‐of‐principle examples of the NIPD of monogenic disorders. However, these approaches can hardly be implemented in clinical practice because they can only distinguish paternally originated alleles.

Maternal plasma contains a mixture of cffDNA and maternal cfDNA while half of the fetal alleles were inherited maternally. Therefore, a quantitative method to count the exact number of different alleles is required to deduce the genotype of a given locus. For this purpose, different approaches were proposed in series studies, including the relative mutation dosage (RMD) approach and the relative haplotype dosage analysis (RHDO).^9^, ^10^, ^11^, ^12^, ^13^, ^14^, ^15^, ^16^, ^17^ The RMD approach directly counted the number of DNA molecules to sort out the ratio of mutant and wild‐type alleles using digital polymerase chain reaction (PCR). The principle of the RHDO approach is to deduce the fetal inheritance of maternally transmitted mutations by quantifying the relative dosages of haplotypes with single nucleotide polymorphism (SNP) alleles in and around the targeted gene. Two modified RHDO approaches, microfluidics‐based linked‐read sequencing technology and targeted locus amplification technology, were reported to be applicable to the NIPD of monogenic disorders. In these two studies, the information of parental haplotypes should first be achieved by sequencing approaches while prior knowledge of proband was not necessary anymore.^18^, ^19^ Overall, the RMD and modified RHDO approaches have proven useful to at‐risk couples of monogenic disorders who may request NIPD for their first‐born children.

Despite proof‐of‐principle studies having clearly showed both clinical feasibility and utility, the leading implementation of NIPD on monogenic disorders is based on PCR methodologies and NGS strategies.^14^, ^15^, ^16^, ^17^, ^18^, ^19^, ^20^, ^21^, ^22^, ^23^, ^24^, ^25^ As PCR‐based approaches usually detect only one or a small number of mutation sites in each reaction, the scarcity of cffDNA hinders its application from larger panels. NGS‐based strategies are mainly used in bespoke and case‐specific tests as they have some inherent limitations, such as a need for prior knowledge of parental mutations or haplotypes, a lack of robustness in the procedure, and the difficulty of scaling up.^14^, ^15^, ^16^, ^17^, ^18^, ^19^, ^24^, ^25^ Although these drawbacks could practically be overcome in research settings, they might set up a hurdle when it comes to clinical application.

We aimed to build a general single‐molecule counting system that could be adapted for gene mutation detection. This system requires accuracy, versatility, a robust yet straightforward procedure, and cost‐effectiveness. Due to the scarcity of cffDNA, precisely counting of the allelic molecules existing in the plasma became the most logical strategy and the ultimate goal.

In this study, we reported a new system termed cfDNA barcode‐enabled single‐molecule test (cfBEST), in which a high portion of allelic molecules could be retrieved and counted to deduce maternal and fetal genotypes. The system contained experimental procedures and in‐house analysis scripts, both of which were robust and versatile enough to allow minor modifications in any new monogenic disorders. In cfBEST, the fraction of fetal DNA could be calculated for a more accurate deduction of maternal/fetal genotypes. The ability of cfBEST to detect low‐abundance mutations was evaluated using cfDNA reference standards and found to be highly consistent with that from droplet digital PCR (ddPCR). In addition, with heterozygous mutation carriers' genomic DNA (gDNA) and cfDNA (whose “true value” mutation ratio is a known number of 50%), the accuracy of cfBEST at counting DNA molecules in which the mutation and wild‐type alleles were of a similar amount could be accessed.

Additionally, we also reported an uncharacterized method that could be used to eliminate the noise sequences caused by the fragmentation of pseudogenes or homologous genes (referred as “noise‐causing genes”) that share high homology with target genes. Clinical validation showed cfBEST to be a reliable system for detecting common mutations for β‐thalassemia on cfDNA samples. This panel for accurate NIPD of β‐thalassemia has been established as a demo, showing the promise of cfBEST to be a universally applicable system for most monogenic disorders.

## 2 Results

### 2.1 Establishment of a cfBEST System to Accurately Count Single Molecules in cfDNA

We developed an NGS‐based methodology of cfBEST to directly deduce the fetal and maternal genotypes by counting single‐allelic molecules and calculating the mutation ratio in cfDNA of maternal circulation without prior knowledge of parental genotypes. If the fetal and maternal genotypes were identical, the mutation allelic ratio should theoretically be 0%, 50%, or 100% in a homozygous normal fetus, heterozygous fetus, or fetus homozygous for the mutant allele, respectively. If the fetal and maternal genotypes were identical in a particular site, the detected mutation ratio represented both genotypes; otherwise the paternally originated fetal allele could have caused an under‐ or over‐representation of the mutant allelic ratio. Therefore, the accurate counting of allelic molecule and calculation of mutation ratio is fundamental in genotype calling. However, this was technically challenging as many factors could skew allelic ratios as conventional amplicon‐based NGS methods inevitably introduce biases by random sequencing errors, nonuniformity of coverage, unbalanced PCR amplification between different alleles, and a potential difference in PCR amplification efficiency between maternal and fetal cfDNA due to their different sizes. Moreover, cfDNA fragmented from noise‐causing genes could be mistakenly aligned as target sequences to skew the mutation ratio. Therefore, a method that could accurately count the real, original, single‐allelic molecules is required.

**Figure 1:**
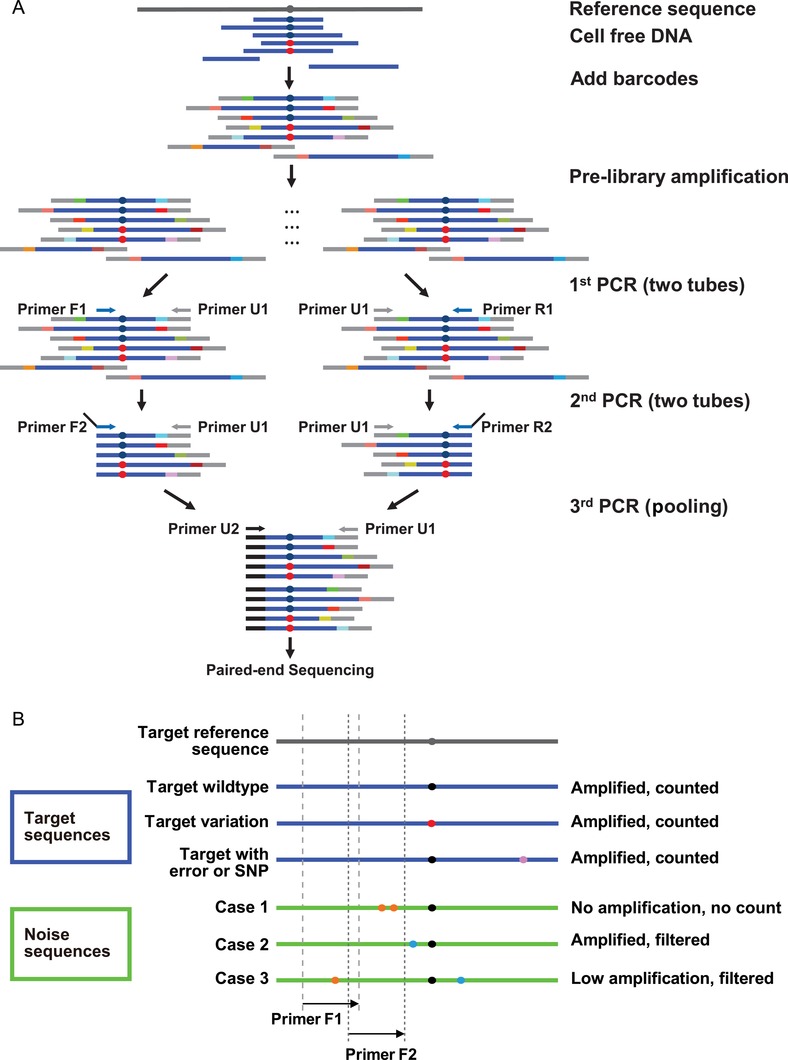
Schematic representation of the cfBEST method. Dark blue and red dots denote wild‐type and mutant sites of interest, respectively. A) The cfBEST protocol. Blue bars denote cfDNA fragments and different colored bars adjacent to the blue bars denote the degenerated barcodes. A prelibrary was built (see the Experimental Section) by amplifying barcoded cfDNA fragments to generate sufficient templates, which was split into two equal portions (referred as “F” and “R”). Each portion was used for the following two PCR reactions: The first PCR with a universal primer (U1) and a target‐specific primer (F1/R1) that was close to the site of interest; the second PCR with the same universal primer (U1) and another primer (F2/R2) containing both a target‐specific part that bound to the region closer to the site of interest than F1/R1 and a universal tail part that was the same as U2. The two portions were pooled together and the third PCR with U1 and U2 was performed for the subsequent massively parallel paired‐end sequencing. In the first and second PCR reactions, two target‐specific primers in the same portion formed a “semi‐nested” PCR to increase the specificity. The design that both primers were bound to near the site of interest could minimize the bias caused by size differences. Each of the barcoded single‐allelic molecules was amplified and sequenced multiple times (reads) and the multiple reads containing the same barcode and breakpoint together were grouped to call a unique original allelic molecule. Therefore, the PCR efficiency did not cause bias, either. B) The strategy for eliminating sequences from pseudogenes or homologous genes (“noise” sequences). The regions flanking the site of interest were analyzed for primer design. A qualified primer was identical to the reference sequence, which was able to amplify the target region (blue lines) without producing noise sequences from other regions (green lines). In most cases, the variations in the primer binding region (orange dots) led to no amplification (case 1); in other cases, there was only one or no variations in the primer binding region, which resulted in an amplified product of noise sequence (case 2) or low‐efficiency amplification (case 3). In order to count reads accurately, a filtering process was designed to eliminate noise sequences. For noise case 1, the PCR did not amplify any noise product. For cases 2 and 3, the unique variation patterns (blue dots) between them and the reference sequence were exploited to filter noise sequences in the bioinformatic analysis step. The sequencing/amplifying error caused by accidental mismatches or SNP (purple dot) in sites different from the variation patterns were allowed. F1/F2 primers are shown as an example in the illustration for one side. For the other side, R1/R2 primers were the same as F1/F2.

In our methodology, cfDNA fragments were ligated with tags containing a 7‐bp degenerated barcode as a UMI (unique molecular identifier) and a universal primer tail (Table S1, Supporting Information). The 7‐bp UMIs, combined with the sequence ends of cfDNA molecules, were used to determine a unique molecule. The use of a relatively small number of prespecified UMIs, in combination with the information of sequence ends, is reportedly adequate to distinguish among different cfDNA molecules in plasma.^26^ Prespecified UMIs have the theoretical advantage of reducing misassignment among indices through sequencing errors and reduced formation of primer dimers during library construction. After barcoding, a prelibrary was built and then divided into two portions for the following steps. The advantage of splitting the prelibrary to conduct a two‐portion PCR amplification symmetrically was that this approach made full use of the original cfDNA molecules. If the site of interest is too close to one end of a cfDNA fragment, sparing no room for the binding of one specific primer, it can still be amplified by the other specific primer from the opposite side. The two sequential PCRs with two partially overlapped primers acted as a semi‐nested PCR to produce more specific target molecules (**Figure** **1**).

When counting single allelic molecules, one significant bias is caused by the noise from the homologous sequences with the target sequences. During evolution, similar sequences, existing as pseudogenes or homologous genes, reside all over the genome due to chromosome repetition, retrotransposition of mature RNAs, and other genetic events.^27^, ^28^ In our methodology, we minimized the influence of noise sequences in three ways. First, for the primer design, we intentionally avoided regions with SNPs, as they could have decreased the PCR efficiency and thus skewed the mutation ratios. Second, the delicate primers were designed in the consideration of both the target gene and the noise‐causing genes. We analyzed the genome and designed the primers (primer F1/F2/R1/R2 in Figure 1) to be identical to the primer‐binding region of the target sequences and to contain at least two mismatches in the 3' end of the primer binding region of the noise sequences. At least two mismatches in the 3' end would guarantee the specificity of the target gene amplification (Figure 1B, case 1). If there were no two close variations between the target and the noise sequences, at least one variation was used as a recognizable marker for the filtering step (Figure 1B, cases 2 and 3). For example, the only variation was intentionally left out of the primer‐binding region (Figure 1B, case 2), as one variation in the primer would not have been sufficient to prevent all nonspecific amplification, and any noise sequence bleeding‐through would have skewed the mutation ratio. Lastly, a bioinformatic filtering algorithm was used to automatically remove all noise sequences. When variations in primers were insufficient in preventing nonspecific amplification of noise sequences, the variation between the target and false sequences was exploited to filter false sequences (Figure 1B, cases 2 and 3).

After the proof‐of‐concept protocol was set up, we evaluated the performance of cfBEST by detecting known low‐abundance mutations in commercial reference standards with corresponding primers (Table S2, Supporting Information). These reference standards served as a valuable testing subject for three reasons: 1) They were commercially available with different mutation ratios. 2) They had a presumed “true value” for their mutation ratios. 3) A ready‐to‐use protocol of ddPCR for these mutation sites existed as a gold standard. We found that cfBEST had comparable performance with ddPCR when detecting samples containing 0%, 0.05%, 0.1%, and 0.5% mutations (Figure S1, Supporting Information).

An accurate fetal DNA fraction is critical for deducing fetal genotype through NIPD. Therefore, we compared cfBEST with the gold standard method for fetal DNA fraction determination. The gold standard measures the relative proportion of mapped Y chromosomal fragments (Y‐assay), which is considered the most reliable assay for fetal DNA fraction determination in NIPT, although it is limited to pregnancies with a male fetus.^29^ In cfBEST, 109 SNPs with high heterozygosity (minor allele frequency, MAF close to 0.5) in the Chinese population were chosen for the calculation of fetal DNA fraction. Using a panel of corresponding cfBEST primers (Table S3, Supporting Information), we applied cfBEST to 26 cases of maternal plasma from pregnancies with karyotyping‐confirmed male fetuses. Y‐assay was conducted accordingly, and the results from cfBEST were highly concordant with those from the Y‐assay (*R* ^2^ = 0.97) (Figure S2, Supporting Information).

### 2.2 Development of a β‐Thalassemia Assay Based on cfBEST

**Figure 2:**
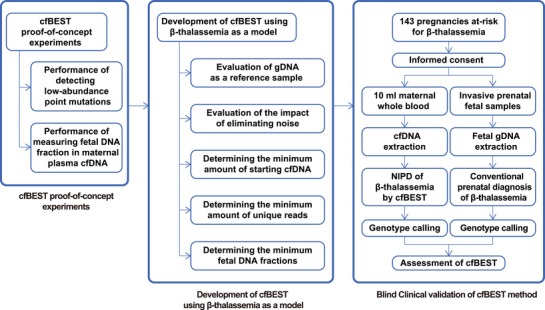
Overview of study design, which was conducted in three stages: a cfBEST proof‐of‐concept experiment, the development of cfBEST using β‐thalassemia as a model, and a blind clinical validation of the cfBEST method.

The high performance of cfBEST in detecting low abundance mutations as ddPCR and measuring fetal DNA fractions as Y‐assay indicated a premise for a scalable method of noninvasively detecting multiple mutations simultaneously. To further demonstrate the potential of cfBEST as a general solution for monogenic disorders, we developed an assay to detect 13 common β‐thalassemia mutation sites in the *HBB* gene. A three‐stage study workflow was designed, including a proof‐of‐concept experiment, assay development using β‐thalassemia as a model, and blind clinical validation (**Figure** **2**). Based on the proof‐of‐concept cfBEST protocol, we designed primers for the following 16 common *HBB* mutations at 13 sites (Figure S3 and Table S4, Supporting Information), which included *HBB*:c.‐79A>G, *HBB*:c.‐78A>G, *HBB*:c.‐78A>C, *HBB*:c.45_46insG, *HBB*:c.52A>T, *HBB*:c.84_85insC, *HBB*:c.126_129delCTTT, *HBB*:c.126_130delCTTT;insA, *HBB*:c.130G>T, *HBB*:c.216_217insA, *HBB*:c.216_217insT, *HBB*:c.79G>A, *HBB*:c.92+1G>T, *HBB*:c.316‐197C>T, *HBB*:c.‐100G>A, *and HBB*:c.315+5G>C.

For a locus in the maternal/fetal cfDNA mixture, there was either one allele (referred to as A) or two alleles (A or B). The maternal genotype could have been either AA (homozygous) or AB (heterozygous). In the AA background, the maternal/fetal combination could have been AAaa or AAab (a and b denote the fetal wild‐type and mutant alleles, respectively). For our molecule counting assay, the accurate determination of the maternal/fetal combination as AAaa or AAab is capable of excellent performance at detecting low‐abundance mutations (Figure S1, Supporting Information). The challenge existed in the AB background of distinguishing ABaa, ABab, and ABbb, in which the mutation ratios were near 50%. The subtle change around 50% could have been from either a true signal caused by the fetal DNA fraction or by sequencing biases. To overcome this challenge, we optimized the β‐thalassemia cfBEST assay (mainly in the AB background) using both cfDNA and genomic DNA from the heterozygous carriers of the corresponding mutations.

**Figure 3:**
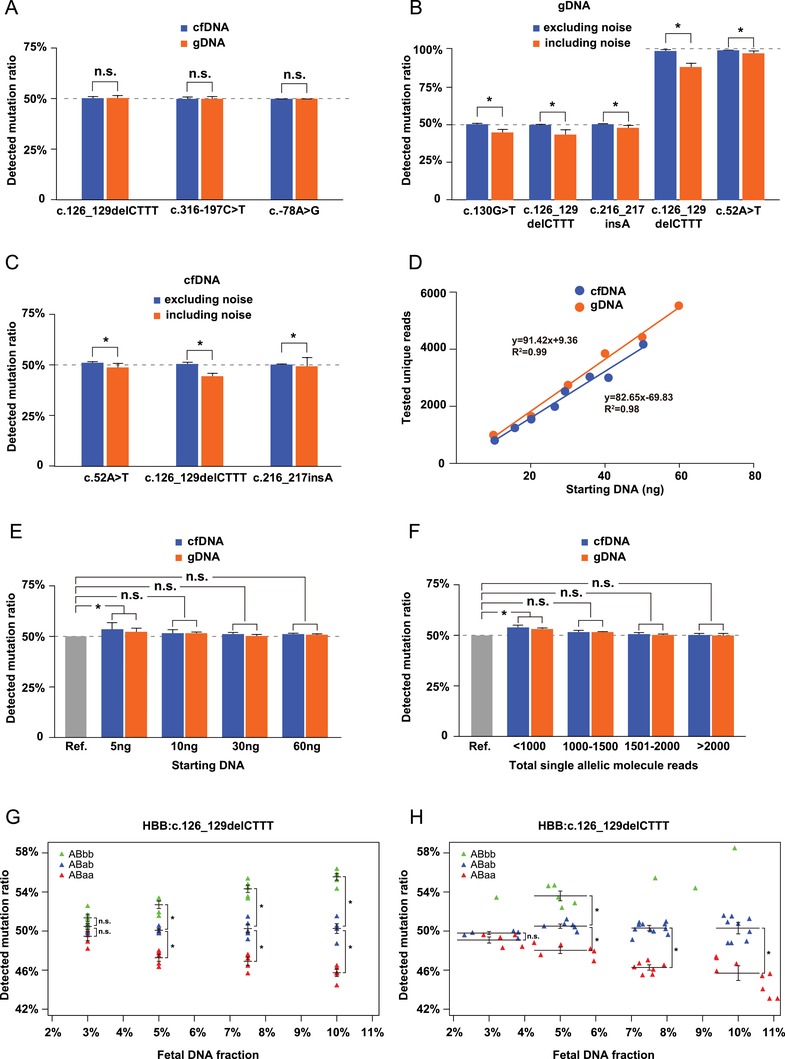
Development and optimization of the cfBEST system using β‐thalassemia as a model. A) Evaluation of gDNA as a reference sample. There was no significant difference between cfDNA and gDNA tested as a reference material with three different types of heterozygous β‐thalassemia mutations. Data are means ± SD; *n* = 5; n.s., not significant (Student *t*‐test). Evaluation of the impact of eliminating noise sequences through primer design and bioinformatic analysis with B) gDNA and C) cfDNA samples. A comparison of the detected mutation ratios between including and excluding noise sequences using B) gDNA samples from three heterozygous and two homozygous β‐thalassemia mutations and C) cfDNA samples from three heterozygous β‐thalassemia mutations. Data are means ± SD; *n* = 5; * *p* < 0.05, n.s., not significant (Student *t*‐test). D) Evaluating the correlation between starting DNA and tested unique reads (Pearson correlation coefficient analysis). E) Determining the minimal amount of starting DNA required for the cfBEST assay. Different amounts of gDNA and cfDNA from a heterozygous carrier of *HBB*:c.79G>A was tested as starting DNA. Data are means ± SD; * *p* < 0.05; *n* = 5; n.s., not significant (Student *t*‐test). All comparison was done between the theoretical value 50% (gray bar, denoted as a reference indicator, Ref.) and the detected ratios. F) Determining the minimal single‐molecule sequencing reads required for the cfBEST assay. The gDNA and cfDNA of a heterozygous carrier for *HBB*:c.79G>A was tested using cfBEST and different depths of sequencing reads were analyzed. Data are means ± SD; * *p* < 0.05; *n* = 5; n.s., not significant (Student *t*‐test). All comparison was done between the theoretical value 50% (gray bar, denoted as a reference indicator, Ref.) and the detected ratios. G) Determining the minimal fetal DNA fraction in maternal plasma required for accurate quantitative genotyping of β‐thalassemia mutations using ultrasonically fragmented gDNA by preparing the mixtures of different ratios. A mixture was made up of the sonicated gDNA from a heterozygous mutation sample that mimicked the background maternal cfDNA (denoted as “AB”) and “fetal” DNA sample (denoted by “aa” for a wild‐type, “ab” for a heterozygote, or “bb” for a homozygote). Four different concentrations of five replicate gDNA samples of ABaa, ABab, and ABbb were applied to cfBEST for mutation ratio detection. Data are means ± SD; *n* = 5. H) A total of 67 samples with *HBB*:c.126_129delCTTT, including 27 cases of ABaa, 31 cases of ABab, and 9 cases of ABbb from the peripheral blood of pregnant women were used to determine the lower limit of fetal DNA fraction. Different concentrations of cfDNA samples of ABaa, ABab, and ABbb were applied to cfBEST for mutation ratio detection. As there were no sufficient ABbb samples for statistics, individual dots denoted the detected ratios. Green triangles denote ABbb, blue triangles denote ABab, and orange triangles denote ABaa in (G,H).

To develop a molecule counting assay and test different conditions for optimization, adequate reference standard material was a necessity. However, cfDNA from heterozygous mutation carriers, whose mutation ratio is known and fixed as 50%, is rare and limited. To overcome the scarcity of cfDNA samples with the desired genotypes, we tested the suitability of genomic DNA (gDNA) of carriers to substitute the corresponding cfDNA for assay optimization. We hypothesized that gDNA, when appropriately fragmented by sonication, could have represented the cfDNA. If this holds true, we will be able to multiple the working materials by over 1000 times from one blood sample, as there is only 2–10 ng cfDNA but 10 µg gDNA in 1 mL of blood on average. Indeed, we applied cfBEST on both cfDNA and gDNA from heterozygous carriers of *HBB*:c.126_129delCTTT, *HBB*:c.316‐197C>T, and *HBB*:c.‐78A>G. There is no significant difference of detected mutation ratio between cfDNA and gDNA, demonstrating the suitability of gDNA as a surrogate of cfDNA in assay development (**Figure** **3**A).

As is described in Figure 1B, one major cause of bias was from the untargeted noise sequences. An optimization process containing the primer design and the bioinformatic filtering was exploited to eliminate noise sequences. To test the influence that noise sequences could potentially exert on the performance of cfBEST, we compared the detected mutation ratios of fragmented gDNA from three heterozygous carriers (mutation ratios = 50%) and two homozygous patients (mutation ratios = 100%). Excluding the noise from total molecule counts was critically important because the noise would have significantly skewed the measurements of both mean values and standard deviations. Before optimization, when the noise sequences were present, cfBEST measurement on gDNA from *HBB*:c.130G>T, *HBB*:c.126_129delCTTT, and *HBB*:c.216‐217insA heterozygotes resulted in a ratio of 44.72% ± 2.28%, 43.19% ± 3.5%, and 47.85% ± 2.71%, respectively. The elimination of the noise improved the measurement to 50.04% ± 1.12%, 49.61% ± 0.82%, and 50.09% ± 0.73%, respectively. For fragmented gDNA from two homozygous genomes of *HBB*:c.126_129delCTTT and *HBB*:c.52A>T, the removal of nontargets improved the mutation ratios of *HBB*:c.126_129delCTTT and *HBB*:c.52A>T from 88.41% ± 2.00% and 96.99% ± 1.78% to 98.90% ± 0.99% and 99.00% ± 0.14%, respectively. This improvement was statistically significant (Figure 3B). We then tested the optimized procedure with cfDNA from three heterozygous carriers of *HBB*:c.52A>T, *HBB*:c.126_129delCTTT, and *HBB*:c.216–217insA. The improvement of the detected mutation ratios from 48.40% ± 2.24%, 43.99% ± 1.90%, and 48.87% ± 4.54% to 50.53% ± 1.01%, 50.16% ± 0.98%, and 49.79% ± 0.70%, respectively, also showed statistical significance (Figure 3C).

The basic principle of the protocol of cfBEST is to label the cfDNA fragments and “read” each sequence with NGS. Losing fragments is inevitable during ligation, cleaning, and transfer. Therefore, the final unique reads only represented a small portion of the total number of fragments in the starting DNA. We hypothesized that with the same sample, the tested unique reads would be correlated with the starting DNA. For both cfDNA and fragmented gDNA from heterozygous *HBB*:c.79G>A, the tested unique reads and starting DNA followed a linear correlation (Pearson correlation coefficient, *R* ^2^ = 0.99 for gDNA, *R* ^2^ = 0.98 for cfDNA) (Figure 3D).

As the tested unique reads were used for calculating the mutation ratios and then deducing genotypes, a certain amount of starting DNA and tested unique reads were required. We used both fragmented gDNA and cfDNA of heterozygous carries to determine the lower limits. For both gDNA and cfDNA from *HBB*:c.79G>A, we found that a limit of 10 ng starting DNA and a minimal of 1000 unique reads are required. We applied 5, 10, 30, and 60 ng of fragmented gDNA with an average size of 166 bp on cfBEST, representing concentrations of 1, 2, 6, and 12 ng cfDNA per mL whole blood respectively. No significant difference was observed in detection of mutation ratios between cfDNA and gDNA. However, when the starting dosage was 5 ng, the detected mutation ratios of cfDNA and gDNA were 53.25% ± 3.18% and 51.48% ± 1.51%, respectively, away from the “true value” of 50% with statistical significance. A starting dosage of 10 ng DNA could meet both the requirement of cfBEST protocol and clinical practice (Figure 3E). Practically, 10 mL maternal blood could contain at least 20 ng cfDNA, which is enough for one test and one backup storage. To provide sufficient statistical power in accurate calculation of mutation ratios, a minimal amount of 1000X total single allelic molecule reads are required for both cfDNA and gDNA (Figure 3F).

The fetal DNA fraction in maternal plasma largely varies from 4% to 25%.^30^ In the background of maternal cfDNA, a subtle change could be due to either a true signal contributed by the fetal alleles or merely sequencing biases. Therefore, the fetal portion should be large enough for an accuracy measurement. We experimentally determined the lower limit of fetal DNA fraction by using artificial mixtures of fragmented gDNA. The sonicated gDNA from a heterozygote (AB) of *HBB*:c.126‐129delCTTT was used as the tested sample containing the “maternal” background, and three different genotypes (aa, ab, and bb) of “fetal” portions. Using four different fetal concentrations (3%, 5%, 7.5%, and 10%), we applied cfBEST to five independent experiments. The detected mutation ratios were plotted for ABaa, ABab, and ABbb. When fetal DNA fraction was at 3%, the data points overlapped, indicating the difficulty of genotype calling based on the mutation ratios. However, when the fetal DNA fraction was at 5% or greater, the data points of the three genotypes were well distinguished (Figure 3G). We applied cfBEST on more gDNA and available cfDNA mixtures in different concentrations and found that the genotypes called by cfBEST showed 100% accuracy when the “fetal” DNA fraction was 5% or higher while it dropped to 40–80% when the fraction comes to 3% (Figure S4, Supporting Information). We used pregnant women's blood to test if 5% fetal DNA fraction was sufficient for genotype calling. To further confirm that 5% fetal DNA fraction was a feasible cutoff, we recruited 67 pregnant women with a heterozygous mutation of *HBB*:c.126_129delCTTT (Table S5, Supporting Information). The maternal/fetal genotypes were determined by conventional invasive molecular diagnosis (IMD), and there were 27 ABaa, 31 ABab and 9 ABbb. We found the fetal DNA fraction of 5% was sufficient to distinguish the genotypes among these 67 cfDNA samples (Figure 3H). Taking 5% as the cutoff would theoretically leads to about clinically acceptable failure rate of 9% based on previous studies. Therefore, 5% was set as the cutoff for our later clinical evaluation.

In summary, the quality control (QC) for cfBEST β‐thalassemia were set as follows as the lower limits: 1) 5% fetal DNA fraction, 2) 10 ng cfDNA, and 3) a minimal of 1000 total unique reads per sample.

### 2.3 Blind Validation in a Cohort of 143 Prenatal Diagnosis Clinical Samples

To validate the clinical utility of cfBEST, we blindly tested 157 plasma specimens from pregnant women carrying an at‐risk singleton fetus and compared the results with a conventional β‐thalassemia molecular diagnosis (Table S6, Supporting Information). There were 157 pregnancy samples that were independently tested by cfBEST and IMD, while the results from the latter are adopted as golden standards. After decoding the achieved samples, the parameters mentioned above (cfDNA > 10 ng, fetal DNA fraction > 5%, reads > 1000) were used for QC. Then reads from samples that passed QC were used to calculate allelic ratio and fetal DNA fraction for deducing the maternal/fetal genotypes.

Among 157 samples, 14 samples failed in QC due to the following reasons: eight contained fetal cfDNA lower than 5%; three samples were excluded for insufficient cfDNA (<10 ng) while the rest three failed with their sequencing unique reads lower than 1000X (Tables S7 and S8, Supporting Information). Overall, 143 out of the 157 fetal/maternal samples were successfully genotyped by both cfBEST and IMD (Table S8, Supporting Information).

To further assess the sensitivity and specificity of cfBEST, the alleles were also introduced in the concordant analysis. A total of 1859 genotype combinations (13 common β‐thalassemia mutation sites in 143 samples) were called. The concordance rate, which is defined as the ratio of 1855 concordant cases among 1859 detected genotypes, was 99.78% (κ = 0.98, Cohen's kappa coefficient) (**Table** **1**). For individual alleles, concordance rates are shown in **Table** **2**. The results showed that the cfBEST assay of β‐thalassemia was highly consistent with those derived from conventional prenatal diagnosis, with a sensitivity of 99.19% (95% confidence interval (CI), 97.62–100%) and a specificity of 99.92% (95% CI, 99.82–100%). Among all alleles, one false negative (0.81%) and three false positive (0.08%) results appeared, resulting in a positive predictive value of 97.62% (95% CI, 94.96–100%) and a negative predictive value of 99.97% (95% CI, 99.92–100%).

**Table 1:**
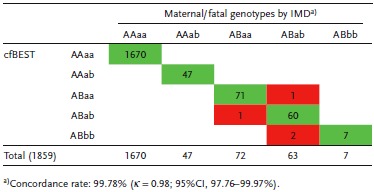
The concordance of maternal/fetal genotype combinations determined by cfBEST with the gold standard IMD. A total of 1859 genotype combinations (13 common β‐thalassemia mutation sites in each of 143 blood samples of pregnant women) were obtained. A and a denote maternal and fetal wild‐type alleles, respectively; B and b denote maternal and fetal mutation alleles, respectively. Green color highlights the cases in which cfBEST and IMD were concordant; red color highlights the cases when cfBEST had errors. Concordance rate is defined as the ratio of 1855 concordant cases among 1859 detected phenotypes; κ is Cohen's kappa coefficient

**Table 2:** Individual allele concordance of the cfBEST test on β‐thalassemia with the gold standard IMD. In the table, a and b denote fetal wild‐type and mutated alleles, respectively. Thirteen out of 16 mutations were listed, as three mutations, *HBB*:c.‐78A>C, *HBB*:c.126_130delCTTT;insA and *HBB*:c.216_217insT, at each of the same sites, were not detected

Mutation ID	cfBEST	IMD		Sensitivity	Specificity
		a	b		
*HBB*:c.315+5G>C	a	286	0		100.00%
	b	0	0		
*HBB*:c.‐78A>G	a	273	0	100.00%	100.00%
	b	0	13		
*HBB*:c.‐79A>G	a	282	0	100.00%	100.00%
	b	0	4		
*HBB*:c.‐100G>A	a	285	0	100.00%	100.00%
	b	0	1		
*HBB*:c.45_46insG	a	286	0		100.00%
	b	0	0		
*HBB*:c.52A>T	a	269	0	100.00%	99.63%
	b	1	16		
*HBB*:c.79G>A	a	285	0	100.00%	100.00%
	b	0	1		
*HBB*:c.84_85insC	a	286	0		100.00%
	b	0	0		
*HBB*:c.126_129delCTTT	a	221	1	98.41%	99.10%
	b	2	62		
*HBB*:c.130G>T	a	285	0	100.00%	100.00%
	b	0	1		
*HBB*:c.216_217insA	a	280	0	100.00%	100.00%
	b	0	6		
*HBB*:c.92+1G>T	a	284	0	100.00%	100.00%
	b	0	2		
*HBB*:c.316‐197C>T	a	269	0	100.00%	100.00%
	b	0	17		
All	a	3591	1	99.19%	99.92%
	b	3	123		

## 3 Discussion

Currently, prenatal diagnosis mainly relies on invasive procedures that pose associated risks to both the fetus and the pregnant mother. The availability of an inexpensive, robust, and easy‐to‐conduct noninvasive prenatal test for monogenic disorders would be advantageous for pregnant women. Here, we reported cfBEST, a straightforward molecular diagnostic technology to detect the maternal/fetal genotypes.

In our study, we built a general single‐molecule counting system for noninvasively detecting monogenic disorders. The system contained an experiment protocol, a panel of SNP primers that were used for fetal DNA fraction determination, a panel of assay‐specific primers, and a set of bioinformatics packages. Ligating cfDNA fragments with UMIs could effectively distinguish PCR duplicates from those original molecules that happened to be fragmented into exactly same starts and ends. According to the sequencing data produced in our study, 2000–3000 unique fragments were recovered per sample while only 200–300 of them are distinguishable by their ends. Therefore, thanks to UMIs, the valid original molecules capacity per sample could theoretically be increased by tenfold for the same amount of sequencing reads without mistakenly filtering them as PCR duplicates. Actually, without UMIs the number of recovered unique reads would be much fewer regardless of the sequencing depths. Moreover, as sequencing cost per gigabyte (GB) goes down with the rapid increase in sequencing throughput, sequencing cost can be further reduced when it is applied on large‐population screening. In addition, various reaction conditions such as ionic strength, annealing temperature, primer design, multiplex combination, PCR additives, and PCR rounds were explored to improve the amplification efficiency and the uniformity of multiplexed PCR, leading to a higher amount of recovered target molecules. The average recovered unique fragments were doubled without increasing sequencing data by introducing the UMI system. Therefore, careful optimization can help reduce the cost of sequencing by at least 50% per sample. The panel of SNP primers for fetal DNA fraction contained 109 oligonucleotides that were designed to amplify the SNPs that were evenly distributed on the genome and that had a MAF close to 0.5. The sequences containing the chosen SNPs encompassed no pseudogenes or homologous genes that could have produced noise sequences capable of skewing the allelic frequencies. A head‐to‐head comparison of cfBEST and ddPCR demonstrated that cfBEST was capable of detecting low‐abundance mutations as well as ddPCR. Another comparison with the gold standard showed a high concordance of the fetal DNA fraction between the cfBEST SNP assay and the Y‐assay. Different from noninvasive prenatal testing for chromosome aneuploidy, which would have a certain number of false positives due to confined placental mosaicism,^31^ our precise counting system that directly quantifies cfDNA allelic molecules could become a diagnostic tool instead of a screening method. Therefore, we referred to cfBEST as an NIPD assay of potential solutions to be applied on monogenic disorders.

We used β‐thalassemia mutations as a model to develop a specific assay based on cfBEST and then tested its clinical validity. We tested the conditions optimal for the β‐thalassemia assay with gDNA from heterozygous carriers, whose mutation ratio is known and fixed as 50%, as they could mimic the sparsity of cfDNA at varying degrees; and then we also compared the detection performance of gDNA and cfDNA with the optimized protocol. All we could rely on is the absolute molecule numbers that reflects the fetal mutation ratios without the prior information of parental genotypes. When maternal genotypes were homozygous (AA), the fetus could either have been (aa) or (ab). Therefore, with a homozygous mother, the technical requirement was similar to that for low‐abundance mutation detection because the presence or absence of the paternal allele (b) could be determined after which the maternal/fetal genotypes could be deduced. A technical challenge existed when the maternal genotype was heterozygous (AB), as slight fluctuation around the maternal allelic ratio (50%) could have been caused by either real signals from fetal cfDNA or mere sequencing biases. To overcome these challenges, we needed to specifically improve the measurement accuracy in detection of heterozygous mutations. In our experiment, we found several ways to improve the measurement accuracy: 1) excluding noise sequences through primer designing and bioinformatics filtering; 2) increasing the starting amount of DNA; 3) increasing the recovered unique reads; and 4) increasing the cutoff of the fetal DNA fraction. We incorporated noise removal into our standard cfBEST protocol, and then experimentally determined the minimal amount of 10 ng DNA, a depth of 1000x unique recovered reads, and the cutoff for a fetal DNA fraction at 5% to be minimum required parameters for clinically acceptable detection.

With these predetermined parameters, we blindly tested 1859 genomes and successfully genotyped 1855 of them (concordance rate 99.78%). The detection of 3718 total alleles specific for each of 13 mutation sites from 143 samples achieved a sensitivity of 99.19% (95% CI, 97.62–100%) and a specificity of 99.92% (95% CI, 99.82–100%). Since we demonstrated cfBEST to be a reliable and accurate method for diagnosing monogenic disorders, we propose guidelines for developing assays based on the cfBEST system. First, a proper SNP primer panel should be designed for accurate estimation of fetal DNA fraction. In our study, this panel was applied to Han Chinese population, so any further development targeting this population could use this panel directly. For other ethnic groups, a panel of primers should be designed on the SNPs with high heterozygosity (MAF close to 0.5) and avoid regions that have noise‐causing genes. Second, specific primers for the mutation sites should be designed to combine with bioinformatic filtering to target the real molecules. Third, the peripheral blood of mutation carries should be collected to extract the gDNA to optimize the corresponding assay. Fourth, the minimal amount of cfDNA, the minimal depth, and the minimal fetal DNA fraction should be experimentally determined by both gDNA and cfDNA. Fifth, a sample cohort should be tested, and sensitivity and specificity should be assessed on allele level.

cfBEST shows equivalent performance with ddPCR in detection of low‐abundance mutations and a high concordance with the Y‐assay in measuring the fetal DNA fraction, which results in both high specificity and sensitivity in the β‐thalassemia assay. Moreover, cfBEST possesses some specific advantages. First, cfBEST has great expansion capabilities. Multiplex PCR assay can reportedly have a room with 640 amplicons in a single test,^32^ and more than 800 amplicons had been achieved in our previous pilot experiment. Low number of SNPs introduced in our method could reduce the cost and allow an accurate genotyping of more loci, which makes it a practical approach for developing simultaneous detection of multiple genetic diseases within one panel. Second, cfBEST needs no prior information of proband or parental genotypes, which were essential for NIPT in previous studies.^18^, ^19^, ^33^, ^34^, ^35^, ^36^, ^37^ More prior information means more costs, more work, and more dropped‐out participants. The molecule counting techniques directly detect the allelic ratio and then deduce the genotypes. Third, cfBEST has a high accuracy for detecting low‐abundance mutations as well as a high precision when the mutation ratio is around 50%, which makes it a general system useful for a variety of molecule counting assays.

To extend the application of cfBEST, some efforts are undertaken to detect both SNVs and copy number variations (CNVs) within one single panel: 1) further enriching fetal DNA from maternally derived fragments according to their differences in size; 2) utilizing the pair‐end split reads to detect deletions or duplications with known breakpoints; 3) In order to detect CNVs with unknown breakpoints, more data should be accumulated to select non‐CNV control regions that share similar PCR amplification efficiency within CNV regions of interest.

In conclusion, a novel approach termed cfBEST was developed for noninvasive prenatal testing of monogenic disorders, and an assay specific to β‐thalassemia was assessed blindly. We managed to correctly genotype 99.78% of fetuses. The detection for alleles achieved a sensitivity of 99.19% (95% CI, 97.62–100%) and a specificity of 99.92% (95% CI, 99.82–100%). Since cfBEST has been demonstrated to be a reliable and accurate method for monogenic disorders, we proposed the guidelines for developing assays for other monogenic disorders. In the future, it can be seen that cfBEST holds the promise of becoming a general and practical system for large‐scale noninvasive molecular screening for various prevalent monogenic disorders for specific population simultaneously.

## 4 Experimental Section

*Study Design*: The study design consisted of three phases: 1) The proof‐of‐concept phase demonstrated that the cfBEST as a molecule counting system could accurately detect low‐abundance mutations in reference standards and determine fetal DNA fractions. 2) The optimizing phase was used to develop a specific assay on cfBEST for β‐thalassemia. 3) The clinical validation phase evaluated the sensitivity and specificity of the β‐thalassemia assay with a cohort of samples.

*Sample and Processing*: The study was approved by the Internal Ethics Committee of Southern Medical University. Blood samples were collected with consent from subjects at the Nanfang Hospital, Southern Medical University, Foshan Maternity & Child Healthcare Hospital, Guangxi Zhuang Autonomous Region Women and Children Care Hospital, and Qinzhou Maternity & Child Healthcare Hospital. These hospitals are located in a region of southern China with a high prevalence of β‐thalassemia.

Samples in the optimization study included both genomic DNA samples from white blood cells and cfDNA samples from plasma. Samples in the optimization study included peripheral blood from nonpregnant β‐thalassemia carriers included the following types: *HBB*: c.126_129delCTTT, *HBB*: c.130G>T, *HBB*: c.216_217insA, *HBB*: c.316‐197C>T, *HBB*: c.52A>T, *HBB*: c.‐78A>G, *HBB*: c.79G>A. The homozygous mutations included four samples: *HBB*:c.126_129delCTTT, *HBB*:c.52A>T, *HBB*:c.316‐197C>T, and *HBB*:c.‐78A>G (Table S5A,B, Supporting Information). A cohort of 26 pregnant women with a singleton male fetus were recruited to draw peripheral blood for the purpose of measuring fetal DNA fraction (Table S3C, Supporting Information). Another cohort of 67 pregnant women with a heterozygous mutation of *HBB*:c.126_129delCTTT were recruited to draw peripheral blood for the determination of the lower limit of fetal DNA fraction (Table S5C, Supporting Information). Samples in the validation study included plasma from 157 pregnant women who were over 18 years old and carried a fetus with a gestational age of 11–24 + 6 weeks (Table S6, Supporting Information). All blood donors had no other conditions except carrying an *HBB* mutation. 10 mL blood samples from pregnant women and β‐thalassemia carriers were collected and processed as described previously.^38^ Briefly, blood was centrifuged at 1600 g, 4 °C for 10 min, and the supernatant was centrifuged for an additional 10 min at 16 000 g to remove cellular debris within 4 h after drawing blood. The buffy coat was used for gDNA extraction, and the cfDNA extraction from stored plasma samples was followed with one more step of 10 min centrifugation at 14 000 rpm to remove precipitates. The sample characteristics are summarized in Table S7 in the Supporting Information. DNA in white blood cells, chorionic villi, and amniotic fluid samples were extracted using the QIAamp DNA Blood Mini Kit (Qiagen), and cfDNA was extracted from plasma using the QIAamp Circulating Nucleic Acid Kit (Qiagen) and quantified through the Qubit® 3.0 Fluorometer (Invitrogen). For IMD, 16 β‐thalassemia mutations at these 13 sites (Figure S3, Supporting Information) were defined by the reverse dot blot assay or Sanger sequencing using a standard protocol in the laboratory.

*Preparation for the Fragmented gDNA as a Standard Reference*: Genomic DNA extracted from white blood cells was fragmented to ≈160 bp by sonication using Covaris M220 (Covaris). Normal healthy individuals, heterozygous carriers, or homozygous patients with β‐thalassemia mutations were recruited to participate the study with informed consent, and each of them donated 20 mL blood except homozygous patients, who provided 2–3 mL leftover samples after the routine blood test.

*NGS Library Construction and Counting Alleles by cfBEST*: cfDNA end repair was performed, and A‐tailing was added using the KAPA Hyper Prep Kit (Kapa Biosystems). T‐tailed DNA was ligated to cfBEST Tag adaptors, and then PCR amplification was performed for ten cycles. The library was then split into two equal portions and separately subjected to two rounds of nested PCR for ten cycles. After Ampure XP bead cleanup, the two portions of PCR product were pooled and used to execute a third PCR for a paired‐end sequencing procedure.

Sequencing libraries were subjected to massively parallel sequencing on the NextSeq CN500 (Illumina) to generate 15 million paired‐end reads (2 × 75 bp) for each sample. The strategy was designed to retrieve all possible allelic fragment templates with different sizes to minimize bias, including some fragments in which the ends were close to the mutation site. As degenerate barcodes were added to cfDNA, the barcode sequence and the adjacent ends of the cfDNA were together used to determine “unique” sequences that were counted to calculate the single‐allele molecules. The final allelic ratio was determined from a minimum of 1000 uniquely barcoded alleles. The bioinformatics analysis was conducted using an in‐house cloud service as previously described.^39^ Further details are provided in the Supporting Information.

*Eliminating Noise Sequences*: The two primers used in the first and second PCR reactions formed a semi‐nested PCR pair referred to as F1/F2 (or R1/R2). In the primer design, the possible “noise‐causing” genes and target genes were analyzed. The primers met these criteria: 1) All primers were designed out of the common SNP sites. 2) At least one of the F1/F2 primers that were identical with the binding region of the target gene had at least two mismatches with all “noise‐causing” genes in the 3' region and one mismatch was within the last five nucleotides. 3) If primers only contained one or no mismatch with the noise‐causing gene, at least one variation in the noise‐causing gene was kept out of the primer binding region, and this variation was included in the PCR product. 4) The variation(s) from (3) was used as the recognizable marker and the bioinformatic filtering step recognized and removed it from the real targets.

*Statistical Analyses*: A student's *t*‐test was applied for all statistical analyses. A *p*‐value below 0.05 was considered to be significant. All data are presented as means ± standard deviation (SD).

## Conflict of Interest

The authors declare no conflict of interest.

## Supporting information

Supplementary

Supplementary
