# Supplementary material for: A Cell‐free DNA Barcode‐Enabled Single‐Molecule Test for Noninvasive Prenatal Diagnosis of Monogenic Disorders: Application to β‐Thalassemia
Source: Adv Sci (Weinh). 2019 Apr 1;6(11):1802332. doi: 10.1002/advs.201802332 (PMC6548944; doi:10.1002/advs.201802332)
Supplement: Supplementary file 1 — Supplementary [file ADVS-6-1802332-s001.pdf]

Copyright WILEY-VCH Verlag GmbH & Co. KGaA, 69469 Weinheim, Germany, 2019.

ADVANCED SCIENCE
Open Access

Supporting Information

for *Adv. Sci.*, DOI: 10.1002/advs.201802332

A Cell-free DNA Barcode-Enabled Single-Molecule Test for Noninvasive Prenatal Diagnosis of Monogenic Disorders: Application to β-Thalassemia

Xingkun Yang, Qinghua Zhou, Wanjun Zhou, Mei Zhong, Xiaoling Guo, Xiaofeng Wang, Xin Fan, Shanhuo Yan, Liyan Li, Yunli Lai, Yongli Wang, Jin Huang, Yuhua Ye, Huaping Zeng, Jun Chuan, Yuanping Du, Chouxian Ma, Peining Li, Zhuo Song, and Xiangmin Xu**

Supplementary Material

Materials

Reference standards were purchased from Horizon Discovery Inc. (Cambridge, UK) and processed according to the manufacturer's manual. The primers (Table S1) used in cfBEST were designed using an in-house algorithm and synthesized by Sangon Biotech, Inc. (Shanghai, China). The cfDNA used for fetal DNA fraction determination was extracted from peripheral blood drawn from pregnant women with a singleton male fetus using the QIAamp Circulating Nucleic Acid Kit (QIAGEN) according to the manufacture's protocol. The concentration of purified cfDNA was measured with the Qubit3.0 dsDNA HS Assay Kit (Thermo Fisher Scientific).

NGS library construction

The NGS pre-library was constructed using the KAPA Hyper Prep Kit (Kapa Biosystems), according to the manufacturer's instructions. DNA molecules were end-repaired with T4 DNA polymerase and Klenow polymerase, and T4 polynucleotide kinase was used to phosphorylate the 5' ends. A 3' overhang was created with a 3'-5' exonuclease-deficient Klenow fragment. Adaptor oligonucleotides, which have a 7 nt barcode, were ligated to the sticky ends (Table S1). The adaptor-ligated DNA was purified directly with 1X Ampure XP beads (Beckman Coulter) to remove un-ligated adapters and adapter dimers. The purified adaptor-ligated DNA was amplified through a 10-cycle PCR using 2× KAPA HiFi HotStart Ready Mix (Kapa Biosystems) with an index primer and a universal primer.

Pre-libraries were split into two equal portions (referred to as “F” and “R”), and each portion was used for two sequential 10-cycles PCR reactions with 2×KAPA HiFi HotStart Ready Mix (Kapa Biosystems). The first round of PCR was performed using a universal primer (U1) complementary to the universal adapter and a target-specific primer (F1 and R1 for the F and R portions, respectively). After Ampure XP bead cleanup, the second PCR was executed using U1 and another target- specific primer (F2 and R2 for F and R portion, respectively). Primer F2 and R2 contained a specific part and a universal tail that was the same as U2. After Ampure XP bead cleanup, the two portions of the PCR product were pooled together to serve as the templates of the third PCR with U1 and U2. Then, the product of the third PCR was used for the following paired-end sequencing procedure. All adaptors and primers used were listed in Table S1.

Determining the low-abundance point mutations through the use of ddPCR and cfBEST

The ddPCR detection was conducted as the users’ manual. Briefly, each sample was added to reaction mixtures that contained 2× ddPCR Mastermix (Bio-Rad) and corresponding TaqMan probes/primers. A 19 uL ddPCR reaction mixture, 6 uL of double distilled water, and 70 uL of droplet generation oil were sequentially loaded into sample wells of a droplet generator cartridge (Bio-Rad). The droplets were manually transferred with care into a 96-well PCR plate, which was then heat-sealed and amplified in a common thermal cycler (Bio-Rad). After PCR, the plate was read on a QX-100 droplet reader (Bio-Rad). Data analysis of the ddPCR was performed with QuantaSoft analysis software (Bio-Rad) according to the manufacturer's manual. For cfBEST detection, three independent

experiments were carried out according to the above protocol. Sequencing data were analyzed, and the mutation ratios were calculated accordingly.

SNPs selection and primer design for fetal determination

For an accurate fetal DNA fraction calculation, included SNP markers were determined through two steps: bioinformatic design and experimental validation. Bioinformatic design was carried out with the following criteria: 1) SNPs were highly heterozygous in the Chinese population, with a MAF close to 0.5; 2) They were distributed in the 22 autosomes evenly (3-5 SNPs per autosome); 3) They avoided regions where CNVs often happened; 4) They were located in a context with moderate GC content. Primers for 144 sites were designed and synthesized. Experimental validation using cfBEST was performed on human genomes carrying the corresponding SNPs. Any primer pairs that resulted in data with a difference greater than 5% between the expected ratio and the detected ratio or with standard deviation (SD) more than 1% in repeated experiment failed our requirements. Among 144 pairs of primers, 35 were filtered. Therefore, 109 SNPs were validated to be suitable for our fetal DNA fraction determination.

Primer design for the β-thalassemia cfBEST assay

Theoretically, four primers should have been designed for each mutation site based on the principle of cfBEST. However, as the 16 common mutations at 13 sites form seven clusters, seven groups of primers were designed. Each group contained four primers, except two groups, which had two primers in one side due to the lack of specific regions suitable for designing primers in the other side (Fig. S3）.

Determining fetal DNA fractions with Y chromosome assay and cfBEST

Determining the fetal DNA fraction using the Y chromosome assay (Y-assay) was conducted in maternal plasma samples from pregnant women with a male fetus. A previously published formula was applied for calculating fetal DNA fraction from Y chromosome sequencing reads (4,5):

%ChrY = (male% ChrY x F) + female% ChrY x (1 - F)

where F = (%ChrY - female%ChrY) / (male% ChrY - female% ChrY)

The fetal DNA fraction measured by the 109-SNP cfBEST assay was calculated by the number of different alleles. If we use A and B to represent the most and second most abundant allele in the plasma, four combinations (AAAA, AAAB, ABAA, and ABAB, the first two letters for maternal and the subscript for fetal) exist. However, only AAAB and ABAA are informative. For AAAB, FF= 2B/(A+B); and for ABAA, FF=(A-B)/(A+B). FF is usually between 4% to 30%, the two combinations can be easily differentiated. SNP outliers ($p < 0.001$) were removed from the detected value through a Chi-squared test, and the maximum likelihood estimation was applied to eliminate non-informative SNPs with borderline maternal/fetal genotypes. The median fetal DNA fraction value of the final data set was defined as the fetal DNA fraction of the sample. The degree of concordance was determined through Person correlation by comparing the performance of cfBEST and Y-assay on 26 samples.

The workflow of bioinformatics analysis

For the cfBEST bioinformatics analysis, a five-step workflow was taken as preprocessing, mapping and filtering, consensus sequence calling, allele counting, and genotyping.

Preprocessing

A raw read sequence consisted of a barcoded cfDNA sequence flanked by two sequencing primers. A total of 1536 barcodes elaborately designed with two or more mismatches among each other were divided into 48 groups in which GC content was exactly balanced on each position, and 96 barcodes in 3 groups were usually adequate to distinguish different single molecules for each sample. The preprocessing of raw sequence included barcode trimming, adapter trimming, and primer recognizing. Initially, the barcode as well as linker sequence was trimmed off the 5' end of a raw read, and reads with barcodes not belonging to the 96 specified barcodes were discarded. Then, the adapter sequence and the barcode sequence on the 3'end of both read ends were recognized and trimmed by detecting the overlap between the two ends with at least 18 nt at a maximum mismatch rate of 0.2. Subsequently, the primer was recognized without any mismatches, and reads with incorrect primers were discarded.

Mapping and Filtering

Preprocessed reads were aligned to hg19 using BWA mem (v0.7.11), and the aligned reads were further filtered according to the following criteria: (i) both read ends had to be mapped; (ii) mapping qualities of both read ends had to be more than 20 so as to filter reads mapped in repeat regions; (iii) the mapped position had to overlap with the target region to filter non-specifically amplified reads; and (iv) the start position of the read had to be identical to the 5' end position of primer.

Consensus Sequence Calling

The pair-end reads with the same barcodes and identical ends were clustered into one family. The families that contained fewer than 4 reads were discarded. On each position of the read ends, the called consensus sequences with more than 2 Ns were discarded if the ratio between the maximal depth and submaximal depth was greater than 2.85, the base with the maximal depth was the same as the base of the consensus sequence, and the consensus base was N

Allele Counting

The consensus sequences were aligned to hg19 using BWA mem (v0.7.11), subsequently sorted by genomic coordinates using samtools (v1.5), and then realigned around indel using GATK IndelRealigner (v3.7). To count the indel allele accurately, the reads around indels were further realigned using an in-house program that mainly modified soft clipping of the indel alignment when the indel was near the end of read. Finally, the depths of alleles at the target loci were counted, and the allele ratio was calculated. Since more than one primer was designed for regions with a high density of target loci, one DNA template could have been amplified by multiple adjacent primers simultaneously and retrieved to derive multiple consensus sequences during consensus calling. To avoid being counted repeatedly, reads amplified by specific primers were assigned to detect specific loci.

Genotyping

The allele count was modeled in a mixture binomial distribution, and the fetal cfDNA fraction was deduced using an EM algorithm with additional 109 SNP loci. The SNP loci with a depth less than 100 were filtered out. With the deduced fetal cfDNA fraction, the posterior probability of maternal-fetal genotype

combinations was calculated using the Bayes formula, and the combination with a maximal posterior probability value was assigned as the final genotyping result for target loci. All these analyses were implemented using an in-house R-script.

Fig. S1

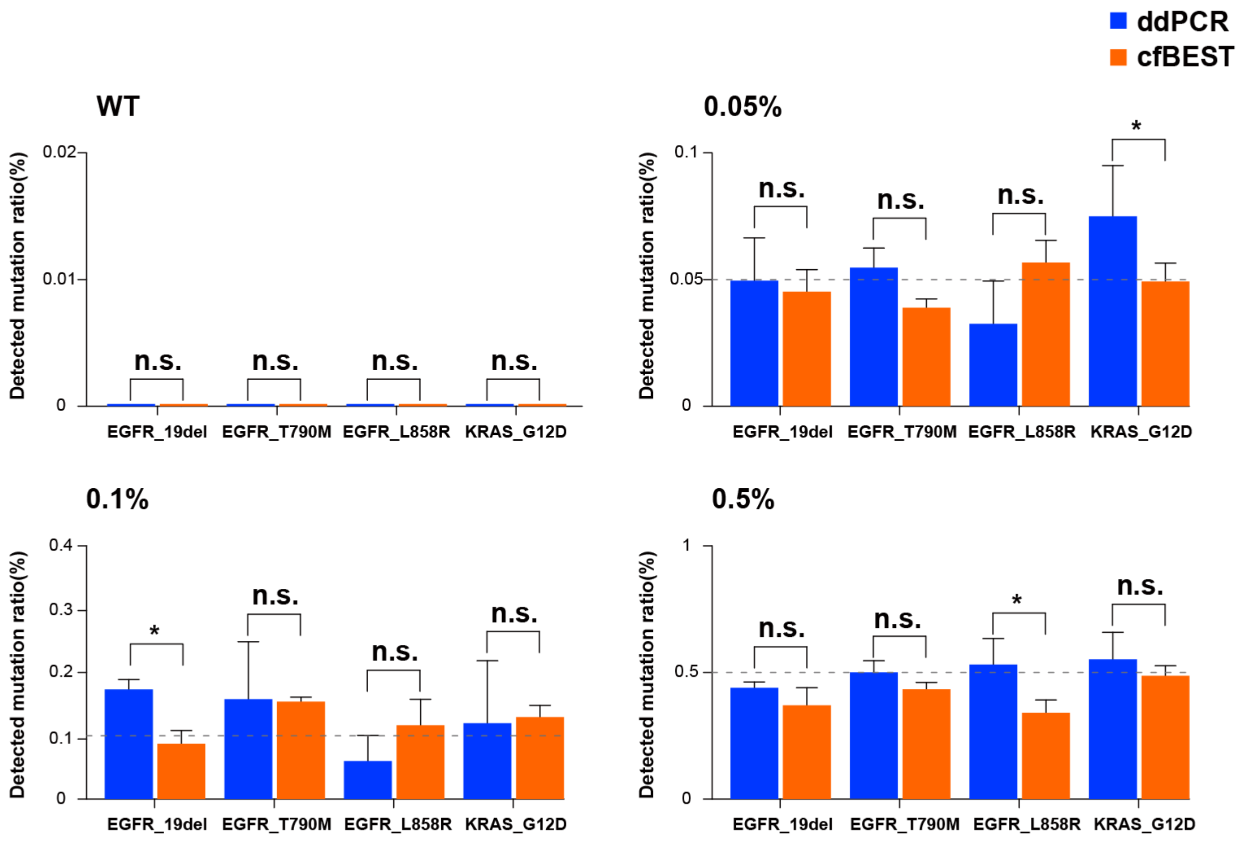

Fig. S1. The cfBEST and ddPCR demonstrated comparable accuracy in detecting low-abundance mutations in reference standards. Four different mutation ratios (0%, 0.05%, 0.1%, and 0.5%) of four reference standards (EGFR_19del, EGFR_T790M, EGFR_L858R, and KRAS_G12D) were subjected to cfBEST and ddPCR. The mutation ratios were then calculated. (* $p<0.05$, t-test).

Fig. S2

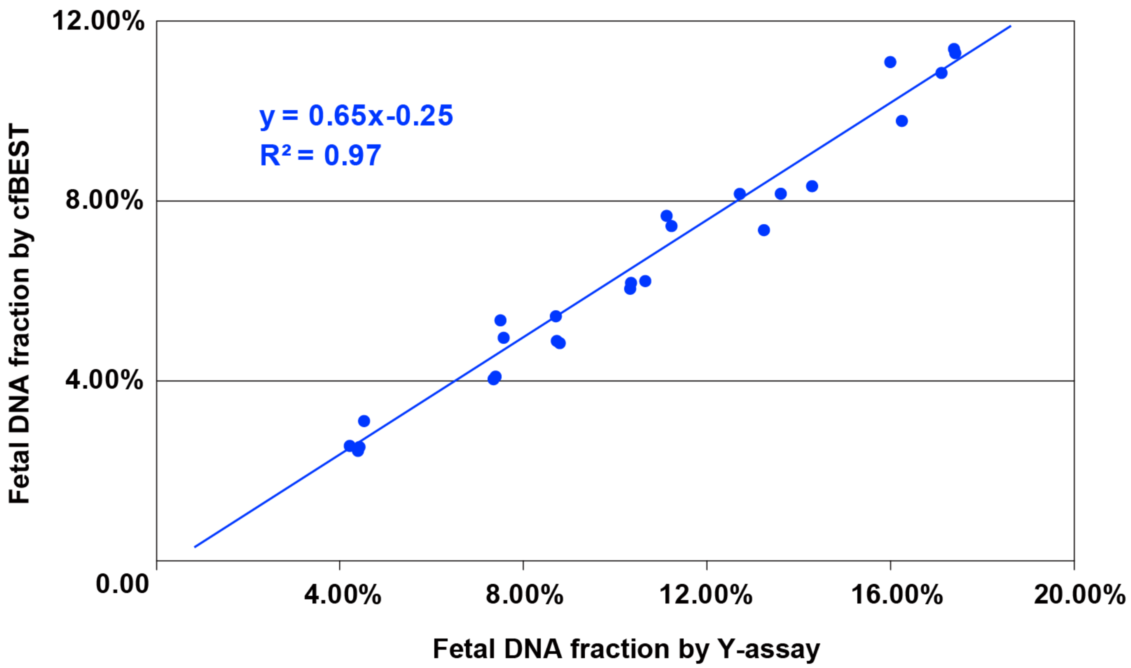

Fig. S2. The cfBEST and Y-assay were highly concordant in fetal DNA fraction determination. Plasma from 26 pregnant women with a singleton male fetus was used for cfDNA extraction, and cfBEST and Y-assay were performed for the fetal DNA fraction determination.).

Fig. S3

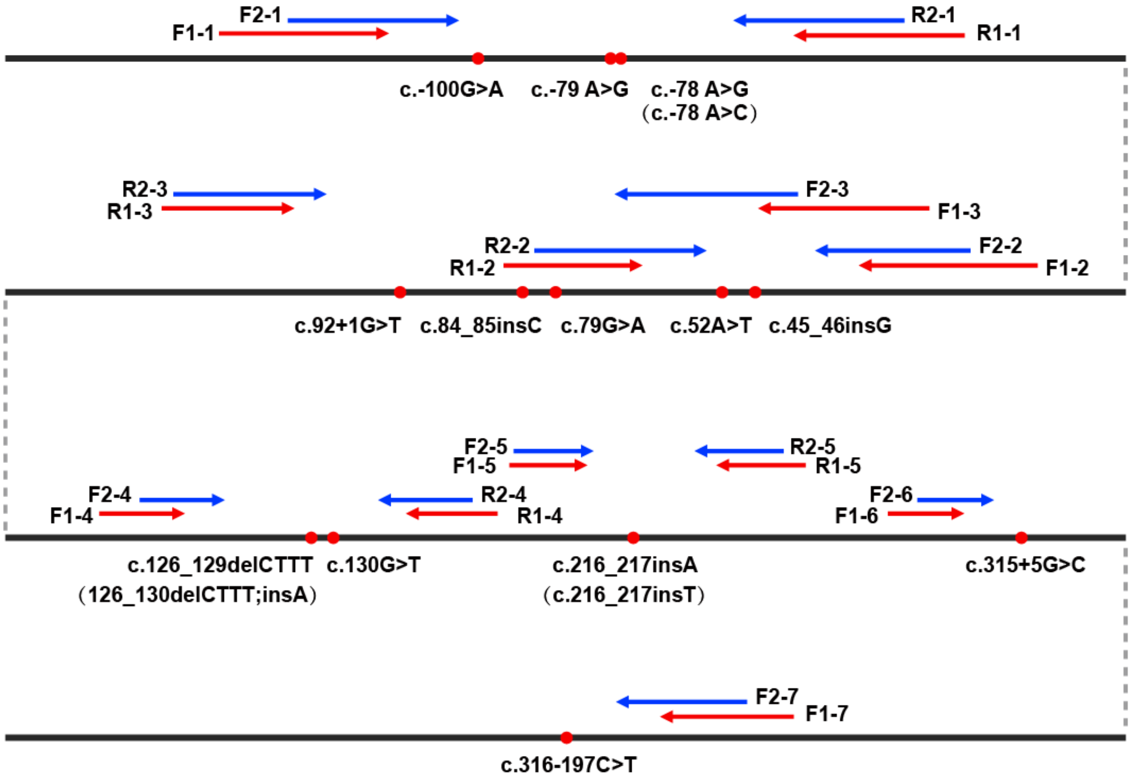

Fig.S3-1

Fig. S3. The schematic representation of primers used for the β-thalassemia cfBEST assay. 16 mutations at 13 sites were grouped into seven groups, each of which required four primers except *HBB*: c.315+5G>C and *HBB*: c.316-197C>T. For these two exceptions, due to the complexity of the surrounding context, only one side was used for primer design. Three mutations, *HBB*:c.-78A>C, *HBB*:c.216_217insT and *HBB*: c.126_130delCTTT;insA, were not detected in these study.

Fig. S4

	HBB: c.126_129delCTTT				*HBB:* c.52A>T				*HBB:* c.-78A>G/C				*HBB:* c.316-197C>T			
"Fetal" %	3.0%	5.0%	7.5%	10.0%	3.0%	5.0%	7.5%	10.0%	3.0%	5.0%	7.5%	10.0%	3.0%	5.0%	7.5%	10.0%
ABaa	40%	100%	100%	100%	60%	100%	100%	100%	80%	100%	100%	100%	40%	100%	100%	100%
ABab	80%	100%	100%	100%	80%	100%	100%	100%	80%	100%	100%	100%	60%	100%	100%	100%
ABbb	60%	100%	100%	100%	80%	100%	100%	100%	80%	100%	100%	100%	80%	100%	100%	100%

Fig. S4. Determining the minimal fetal DNA fraction available for accurate quantitative genotyping of β-thalassemia mutations using physically fragmented gDNA by preparing the mixtures of different ratios. A mixture contained a majority of a heterozygous mutation sample which mimicked the background maternal cfDNA (denoted as "AB") and "fetal" DNA sample (denoted by "aa" for a wild-type, "ab" for a heterozygote, or "bb" for a homozygote). Five replicate samples were applied to cfBEST for genotype calling. The percentage of certain combined maternal-fetal genotypes represents the accuracy of the genotype determined by cfBEST. Green denotes 100% accuracy, and red denotes partial accuracy.

Tables and additional files that cannot be embedded into the Word file are included with the submission. These include:

Table S1. Sequences of adaptors with UMIs and primers used in cfBEST.

Table S2. Primers used for detecting low-abundance mutations in cfBEST.

Table S3. Primers and individuals' information used for fetal DNA fraction determination.

Table S4. Primers used for β-thalassemia in cfBEST.

Table S5. Information about individuals whose DNA was used in the optimization study.

Table S6. Information about individuals whose DNA was used in the validation study.

Table S7. Validation study samples characteristics.

Table S8. Summary of all of the subjects in the validation study.

Data file S1. The NGS sequencing data.

Data analysis package S1.
